# Associations of Childhood and Adulthood Cognition with Bone Mineral Density in Later Adulthood: A Population-Based Longitudinal Study

**DOI:** 10.3389/fnagi.2017.00241

**Published:** 2017-07-25

**Authors:** Rebecca Bendayan, Diana Kuh, Rachel Cooper, Stella Muthuri, Graciela Muniz-Terrera, Judith Adams, Kate Ward, Marcus Richards

**Affiliations:** ^1^MRC Unit for Lifelong Health and Ageing, University College London London, United Kingdom; ^2^Centre for Dementia Prevention, University of Edinburgh Edinburgh, United Kingdom; ^3^Radiology and Manchester Academic Health Science Centre, Manchester Royal Infirmary, Central Manchester University Hospitals, NHS Foundation Trust Manchester, United Kingdom; ^4^Centre for Imaging Sciences, Faculty of Biology, Medicine and Health, University of Manchester Manchester, United Kingdom; ^5^Nutrition and Bone Health, MRC Elsie Widdowson Laboratory Cambridge, United Kingdom; ^6^MRC Lifecourse Epidemiology Unit, University of Southampton Southampton, United Kingdom

**Keywords:** aging, bone health, bone mineral density, cognitive performance, life course

## Abstract

This study explores the association between cognitive ability in childhood and midlife and bone health outcomes in early old age; and the relationships of these bone measures with contemporaneous and subsequent cognitive ability in the MRC National Survey of Health and Development (NSHD). This British birth cohort assessed areal and volumetric bone mineral density (aBMD and vBMD) at age 60–64, derived from peripheral quantitative computed tomography and dual-energy X-ray absorptiometry, and cognitive performance from childhood to age 69, among 866 women and 792 men. Cognitive performance at age 15 was assessed using tests of verbal and non-verbal ability, and mathematics; and memory and search speed tasks were administered at ages 53, 60–64, and 69. Covariates included body size, pubertal timing, smoking, leisure time physical activity, socioeconomic circumstances and menopause timing. Multiple linear regression analyses showed that higher childhood cognitive ability was associated with higher hip aBMD, in women, and greater cortical and trabecular vBMD, in men. For women, there were positive associations between hip aBMD and total vBMD, and contemporaneous cognitive ability with associations also extending to subsequent cognitive ability for total vBMD. For men, some associations with trabecular and total vBMD emerged at ages 60–64 and 69 but only after adjusting for education, occupational class and health behaviors. Our findings highlight that higher cognitive ability in childhood is associated with BMD in early old age and these associations might be explained by social and behavioral pathways. The results suggest that individuals with greater cognitive ability in early life are more likely to engage in healthy behaviors (e.g., leisure time physical activity) in adulthood, which in turn are associated with greater BMD later in life. Associations between bone health and cognitive performance should be considered within a life course framework; and the potential role of smoking and physical activity should be addressed when advising adults at high future risk of osteoporosis and fracture.

## Introduction

Low bone mineral density (BMD) is a predictor of fracture in men and women (Marshall et al., 1996; Melton et al., 1998; Bliuc et al., 2015), which in turn is associated with mortality and disability among older adults (Johnell et al., 2004; Haentjens et al., 2010; Frost et al., 2013). Moreover, research to date has found that there is an association between cognitive impairment and risk of fracture (Egan et al., 2007); one possible mechanism is via low BMD, which is associated with poorer cognitive function and elevated risk of dementia in older adults (Yaffe et al., 1999; Zhang et al., 2001; Lui et al., 2003; Brownbill and Ilich, 2004; Tan et al., 2005; Zhou et al., 2011; Lee et al., 2012; Sohrabi et al., 2015).

To date, the direction of the association between BMD and cognitive function remains unclear. The cited studies measured BMD simultaneously or before assessing cognition, raising the suggestion that lower BMD could lead to poorer cognitive performance or greater subsequent cognitive decline. However, BMD and bone loss are unlikely to affect cognition directly (Yaffe et al., 1999; Lui et al., 2003); BMD is more likely to reflect a wider range of health-related aging processes; and these associations might operate in the reverse direction through behavioral factors (Yaffe et al., 1999); or they may share common physiological causes such as estrogen exposure and physical frailty.

The MRC National Survey of Health and Development (NSHD), the oldest of the British birth cohort studies assessed areal and volumetric bone mineral density (aBMD and vBMD) at age 60–64, and cognitive performance in childhood, midlife and most recently at age 69. It therefore provides a unique opportunity to investigate: (a) whether prior measures of cognitive performance in childhood and midlife are associated with BMD at ages 60–64, taking account of body size, pubertal timing, adult lifestyle, socioeconomic circumstances, and menopause timing (main aim 1); and (b) whether BMD is associated with cognitive performance at ages 60–64 and 69 taking account of prior cognition, and other covariables (main aim 2).

## Materials and methods

### Sample

The NSHD is a cohort study of 2,815 males and 2,547 females followed up since their birth in 1 week in March 1946 in England, Scotland and Wales. At the 23rd follow up when study members were aged between 60 and 64 years, 2,856 who were still alive and had a known current address in mainland Britain were invited for assessment at one of six clinical research facilities (CRF); those unable or unwilling to travel were offered a home visit by a research nurse (Kuh et al., 2011). Of these, 2,229 (78%) underwent assessment: 1,690 attended a CRF and the remaining 539 were seen in their homes (Stafford et al., 2013). Of the remaining participants, 778 had died, 570 were living abroad, 594 had previously withdrawn from the study and 564 were lost to follow-up. Relevant ethical approval was received for each assessment; in 2006–2010 this was obtained from the Central Manchester Local Research Ethics Committee (07/H1008/245) and the Scotland A Research Ethics Committee (08/MRE00/12) and in 2014–2015 from the Queen Square Research Ethics Committee (14/LO/1073) and the Scotland A Research Ethics Committee (14/SS/1009). All participants gave written informed consent.

### Bone health assessment at 60–64 years

Of those attending a CRF, 792 men and 866 women underwent whole body, proximal femur and lumbar spine dual-energy X-ray absorptiometry (DXA) scans and 658 men and 697 women additionally underwent a peripheral quantitative computed tomography (pQCT) scan of the non-dominant radius. DXA scans were acquired in all 6 clinical research facilities using the QDR 4500 Discovery (Hologic Inc., Bedford, MA) with five sites also collecting pQCT data using XCT 2000 (Stratec, Pforzheim, Germany) scanners. Details of scan acquisition and cross-calibration have been previously described (Kuh et al., 2014). Standard manufacturer protocols were followed for data analysis. Variability between centers was monitored for the DXA scanners using the European Spine Phantom and for the pQCT scanners using the European Forearm. Standard manufacturer procedures were followed for daily quality assessment and control and all phantom and scan analysis were centralized to one center (JEA) for grading, analysis and collation of a harmonized database. Repeat precision was determined in one center and was <1% for DXA measurements and for pQCT ranged between 1 and 3%. For comparison with previous research (Yaffe et al., 1999; Lui et al., 2003; Sohrabi et al., 2015) and to assess the most clinically relevant skeletal sites, the bone outcomes for this analysis were DXA derived measurements of lumbar spine (L1–L4) and total hip areal aBMD and pQCT measures of trabecular, cortical volumetric vBMD at 50% radius site and total and trabecular vBMD at the distal 4% radius site.

### Cognitive performance over the life course

At age 15, tests of verbal and non-verbal ability (AH4), reading comprehension (Watts-Vernon) and mathematics designed especially for the study by the National Foundation for Educational Research in England and Wales, were administered (Pigeon, 1968). An overall score of general cognitive ability was generated as the sum of the standardized scores on these tests, re-standardized to a mean of 0 and standard deviation of 1.

At ages 53, 60–64, and 69 cognitive tests were administered by nurses following standardized training and protocols (Richards and Sacker, 2003). At each age, memory was measured using a 15-item word-learning task. The overall score was the total number of words correctly recalled after three trials (maximum score = 45). Two different word lists were alternated between assessments to minimize practice effects. Speed of processing was assessed using a timed letter search task which required participants to cross out the letters P and W, randomly embedded within a grid of other letters, as quickly and accurately as possible in 1 min. The overall score represents the total number of letters searched (maximum score = 600). These scores were also standardized for comparative purposes.

### Covariables

Based on previous analyses in NSHD (Kuh et al., 2011, 2016a), and previous research on cognition and bone health (Yaffe et al., 1999; Zhang et al., 2001; Lui et al., 2003; Brownbill and Ilich, 2004; Tan et al., 2005; Zhou et al., 2011; Lee et al., 2012; Sohrabi et al., 2015), the following potential confounders or mediators of the association between cognitive performance and bone health were selected: body size at 60–64 was assessed by height (m) and weight (kg), according to standard protocols. For the purposes of analyses both were sex-standardized (to a mean of 0 and *SD* of 1) within the sample who were scanned. Pubertal timing was assessed using the timing of peak height velocity (height tempo) derived using the Superimposition by Translation and Rotation (SITAR) model (Cole et al., 2016). A negative height tempo indicates earlier puberty relative to the study population mean. Other covariables included were: educational attainment by 26 years, classified into below ordinary secondary qualifications (none/vocational), ordinary secondary qualifications (“O” levels and their equivalents), advanced secondary qualifications (“A” levels and their equivalents), and higher qualifications (degree or equivalent); occupational class at age 53 (or if not available, the most recent previous measure in adulthood) categorized as manual or non-manual according to the Registrar General's social class classification (Galobardes et al., 2006); smoking status at age 60–64 (smoker or non-smoker); and participation in leisure time physical activity at age 60–64 (active or inactive in the last month). In addition, for women, age of period cessation and menopause type (natural menopause, bilateral oophorectomy, hysterectomy), defined using reports at multiple follow-ups were also considered (Kuh et al., 2016b).

### Statistical analyses

To address the first main aim, we tested associations between childhood cognitive ability, search speed and verbal memory at age 53 with each measure of aBMD and vBMD, using a series of standard multiple linear regression analyses. Natural logarithms for all bone variables were used. The coefficients from these models are presented as the percentage difference in the bone outcome by category (%), or per unit increase (B; Cole, 2000). Cole (2000) provided a theoretical and practical case for using the natural log scale, multiplied by 100, to calculate and present percentage differences. These are especially useful for comparative purposes and facilitate the interpretation of the results in models with different bone outcomes. All models were adjusted for current body size (height and weight at 60–64 years), and then additionally adjusted for timing of puberty (height tempo), occupational class, current smoking, and leisure time physical activity. In addition, sensitivity analyses including educational attainment were performed. For example, the fully adjusted theoretical model for bone health outcomes is defined as:

$$\begin{array}{l} BMD_{i}=\beta_{0}+\beta_{1}cogn15_{i}+\beta_{2\;}height_{i}+\beta_{3\;}weight_{i}\\ \;+\beta_{4\;}height\_tempo_{i}+\beta_{5\;}occupational\_class_{i}\\ \;+\beta_{6\;}smoking_{i}+\beta_{7\;}physical\_activity_{i}\;+\;\varepsilon_{i\;} \end{array}$$

To address the second main aim, we tested the cross-sectional associations between BMD and verbal memory and search speed at age 60–64, then prospective associations with the same cognitive measures at age 69. Statistical models were similar to those used to test the first main aim, except that cognitive scores at age 69 were additionally adjusted for their corresponding scores at age 60–64, and educational attainment was included but not pubertal timing. For these analyses, bone measures were standardized in the same way as the cognitive measures.

In additional analyses, which aimed to examine whether any associations between BMD and verbal memory and search speed at ages 60–64 and 69 among women were explained by menopause timing (Kuh et al., 2016b), a series of standard multiple linear regression analyses were run on the sub-sample of women with data on age of period cessation (in months since birth) and type of menopause.

Missing data on health behaviors and socioeconomic factors were imputed to maximize the analytical sample, with multiple imputations by fully conditional specification via Markov Chain Monte Carlo (MCMC) performed; all analyses presented were conducted across 30 imputed data sets and combined using Rubin's rules, according to the percentages of missing data in each model (Dong and Peng, 2013). When imputing missing data we fill in missing values multiple times using the information contained in the observed data, and within the imputation methods, multiple imputation acknowledges the uncertainty associated with imputed values (Little and Rubin, 2002) by first imputing missing data multiple times, in the present study 30 times to produce 30 complete datasets; then, it analyses each dataset and combines the 30 results, pooling them to provide a single estimate of the parameter. When the missing data patterns are arbitrary, traditional predictive models are difficult to develop to estimate these missing values, and simulation methods, as MCMC, are needed. In the present study, a fully conditional specification via MCMC was adopted. This semi-parametric alternative specifies the multivariate model by a series of conditional models, one for each incomplete variable, preserving the unique distributional characteristics of the data. All statistical analyses were performed using SPSS 22.0 for Windows Software (SPSS Inc.).

## Results

Women had higher mean verbal memory and search speed scores at each age but these means showed an overall pattern of age-related decline for both sexes (Table 1). Women were shorter and lighter than men and had lower cortical vBMD at the 50% radius site, total and trabecular vBMD at the 4% radius site and total hip and lumbar spine aBMD. Given the sex differences in the bone measures and the known sexual dimorphism in the timing of age-related versus menopausal bone loss and in fracture risk, separate models for women and men were conducted.

**Table 1:** Descriptive statistics for each bone health variable and covariates for men and women's maximum sample.

	**Women**		**Men**	
	***N***	**Mean *(SD)***	***N***	**Mean *(SD)***
**COGNITIVE VARIABLES**				
**Memory at age**				
53^*^	827	26.24 (5.89)	730	24.31 (5.96)
60–64^*^	849	29.97 (5.99)	772	23.65 (5.85)
68–69^*^	740	23.89 (5.84)	679	21.91 (5.83)
**Search speed at age**				
53	827	292.47 (74.11)	737	281.50 (75.17)
60–64^*^	849	273.67 (67.43)	777	268.04 (72.27)
68–69^*^	744	270.61 (71.41)	687	262.23 (72.43)
**BONE VARIABLES AT AGE 60–64**				
**pQCT- 50% diaphyseal radius**				
Cortical vBMD (mg/cm^3^)^*^	695	1148.15 (39.33)	655	1158.54 (34.87)
**pQCT- 4% distal radius**				
Trabecular vBMD (mg/cm^3^)^*^	685	171.68 (42.07)	652	205.47 (42.20)
Total density vBMD (mg/cm^3^)^*^	686	329.47 (70.54)	654	390.30 (66.66)
**DXA aBMD (g/cm2)**				
Spine L1-L4 aBMD^*^	861	0.94 (0.16)	788	1.05 (0.18)
Total Hip aBMD^*^	853	0.86 (0.13)	778	1.00 (0.14)
**COVARIABLES**				
Height (m)^*^	861	1.62 (0.05)	788	1.75 (0.06)
Weight (kg)^*^	861	72.28 (0.48)	788	85.15 (12.94)
SITAR height-tempo	861	−0.005 (0.05)	788	−0.0002 (0.05)
Education^*^	856		745	
Below ordinary	231	28.3%	208	27.9%
Ordinary secondary	267	32.6%	148	18.8%
Advanced secondary	257	31.4%	241	30.6%
Degree	63	7.7%	148	18.8%
Occupational Class (Manual)^*^	860	19.4%	788	27.4%
Smoking (Current)	856	9.9%	779	10.8%
Physical activity (inactive in the last month)^*^	835	42.4%	737	38.8%
Type of menopause	705			
Natural/HRT	516	73.20%		
Age of period cessation (years)		52.01 (3.72)		
Hysterectomy/bilateral oophorectomy	189	26.80%		
Age of period cessation (years)		44.53 (6.53)		

* *Significant differences between men and women at p < 0.05*.

### Childhood cognitive ability and BMD at age 60–64

Among women, higher childhood cognitive ability was associated with higher hip aBMD; a one standard deviation (*SD*) increase in cognitive ability at age 15 was associated with a 1.1% greater (95% CI 0.1−2.1%) hip aBMD after adjustment for body size (Table 2). This estimate was slightly weakened by adjustment for height tempo, and was fully attenuated after additional adjustments for smoking, leisure time physical activity, and occupational class (Table 3).

**Table 2:** Percentage differences in cortical, trabecular and total vBMD and lumbar spine and total hip aBMD at 60–64 years per 1 *SD* increase in each cognitive score, adjusted for current height and weight.

	**pQCT Measures**												**DXA Measures**							
	**pQCT- 50% radius (mg/cm**^**3**^**)**				**pQCT-4% radius (mg/cm**^**3**^**)**								**DXA aBMD (g/cm**^**2**^**)**							
	**Cortical vBMD**				**Trabecular vBMD**				**Total Density vBMD**				**Spine L1–L4 aBMD**				**Total Hip aBMD**			
	***N***	**%**	**95% CI**	***P***	***N***	**%**	**95% CI**	***p***	***N***	**%**	**95% CI**	***p***	***N***	**%**	**95% CI**	***p***	***N***	**%**	**95% CI**	***p***
**WOMEN**																				
Cognition 15	577	0.1	−0.2 to 0.4	0.56	569	−0.4	−2.5 to 1.6	0.68	569	−0.3	−2.1 to 1.4	0.72	709	1	−0.3 to 2.2	0.12	702	1.1	0.1 to 2.1	0.02
Search Speed 53	577	−0.1	−0.4 to 0.1	0.30	569	−1.5	−3.5 to 0.6	0.15	569	−0.6	−2.4 to 1.1	0.47	709	−0.9	−2.1 to 0.3	0.16	702	−0.2	−1.1 to 0.8	0.72
Memory 53	576	−0.1	−0.4 to 0.2	0.48	568	−0.8	−2.9 to 1.3	0.44	568	−0.9	−2.7 to 0.8	0.29	709	0.4	−0.8 to 1.6	0.53	702	0.3	−0.6 to 1.3	0.50
**MEN**																				
Cognition 15	527	0.6	0.3 to 0.9	<0.001	524	1.9	0 to 3.8	0.05	526	0.3	−1.2 to 1.8	0.67	633	0.8	−0.5 to 2.1	0.21	625	0.4	−0.6 to 1.5	0.40
Search Speed 53	527	0.2	−0.1 to 0.4	0.21	524	2.1	0.2 to 4	0.02	526	1.2	−0.3 to 2.7	0.12	633	0.8	−0.5 to 2.1	0.21	625	0.5	−0.6 to 1.5	0.37
Memory 53	522	0.2	0 to 0.5	0.09	519	0.2	1.7 to 2.2	0.83	521	−0.6	−2.2 to 0.9	0.43	627	0.5	−0.8 to 1.7	0.45	619	0.01	−1 to 1	0.97

**Table 3:** Percentage differences in cortical and trabecular vBMD and total hip aBMDat 60–64 years per 1 *SD* increase in the cognitive scores at age 15 and 53, adjusted for body size and additionally adjusted for height tempo, adult health behaviors and later (at age 53) and prior cognition (at age 15), respectively.

	**pQCT Measures**								**DXA Measures**			
	**pQCT- 50% radius (mg/cm**^**3**^**)**				**pQCT-4% radius (mg/cm**^**3**^**)**				**DXA aBMD (g/cm**^**2**^**)**			
	**Cortical vBMD (mg/cm**^**3**^**)**				**Trabecular vBMD (mg/cm**^**3**^**)**				**Total Hip aBMD**			
	***N***	**%**	**95% CI**	***p***	***N***	**%**	**95% CI**	***p***	***N***	**%**	**95% CI**	***p***
**WOMEN**													
**Cognition 15**													
Additionally adjusted for SITAR height-tempo	577	0.1	−0.2 to 0.4	0.56	569	−0.8	−2.9 to 1.3	0.43	702	1	0 to 2	0.04
Additionally adjusted for Occupational class, smoking and physical activity	577	0.01	−0.3 to 0.3	0.96	569	−1	−3.2 to 1.2	0.37	702	0.8	−0.3 to 1.8	0.15
Additionally adjusted for Memory at age 53	577	0.01	−0.3 to 0.3	0.90	569	−0.8	−3 to 1.4	0.48	702	0.8	−0.2 to 1.8	0.12
**MEN**													
**Cognition 15**													
Additionally adjusted for SITAR height-tempo	527	0.6	0.3 to 0.8	<0.001	524	1.5	−0.4 to 3.4	0.12	625	0.3	0.7 to 1.3	0.56
Additionally adjusted for Occupational class, smoking and physical activity	527	0.5	0.2 to 0.8	0.001	524	0.9	−1.3 to 3.1	0.43	625	0.7	−0.5 to 1.9	0.23
Additionally adjusted for Memory at age 53	527	0.5	0.2 to 0.8	0.002	524	0.7	−1.5 to 2.9	0.54	625	0.7	−0.5 to 1.9	0.27
**Search speed at age 53**													
Additionally adjusted for SITAR height-tempo	527	0.2	−0.1 to 0.4	0.2	524	2.1	0.2 to 4	0.02	625	0.4	−0.6 to 1.5	0.39
Additionally adjusted for Occupational class, smoking and physical activity	527	0.01	−0.2 to 0.3	0.75	524	1.4	−0.5 to 3.4	0.13	625	0.4	−0.6 to 1.5	0.40
Additionally adjusted for Cognition 15	527	0.01	−0.3 to 0.3	0.92	524	1.4	−0.6 to 3.3	0.16	625	0.4	−0.7 to 1.4	0.47

For men, higher childhood cognitive ability was associated with greater cortical and trabecular vBMD (Table 2). The body size adjusted estimate for cortical vBMD (0.6%, 95% CI 0.3−0.9%) was not affected by further adjustment for height tempo, and remained, albeit slightly weaker, after additional adjustments for smoking, physical activity, and occupational class (Table 3). The body size adjusted estimate for trabecular vBMD (1.9%, 95% CI 0.01−3.8%) was slightly attenuated after adjusting for height tempo, but was no longer evident after further adjustments for smoking, physical activity, and occupational class (Table 3).

Although there were no other significant associations between childhood cognitive ability and BMD in men or women before or after adjustments, the direction of the associations with the other bone outcomes was consistent (Tables 2, 3). Sensitivity analyses revealed that the associations between childhood cognitive ability and specific bone outcomes remained after additionally adjusting for educational attainment.

### Verbal memory and search speed at age 53 and BMD at age 60–64

In women, there were no associations between verbal memory or search speed at age 53 and BMD at ages 60–64 (Table 2).

In men, higher scores in search speed were associated with greater trabecular vBMD; 1*SD* increase in search speed was associated with a 2.1% (95% CI 0.2−4%) difference in trabecular vBMD, after adjustment for body size (Table 2). Further adjustments for height tempo did not alter this association but the association was no longer significant after additional adjustments for smoking, physical activity, educational attainment, and occupational class were made (Table 3).

### BMD at age 60–64 and verbal memory and search speed at ages 60–64 and 69

In women greater total vBMD was associated with faster search speed after adjustment for body size (Table 4). Further adjustments for educational attainment, smoking, physical activity, and occupational class slightly strengthened these associations (Table 5). For search speed at 69, the association with total vBMD remained, albeit somewhat attenuated, after additionally adjusting for search speed at 60–64.

**Table 4:** Standardized verbal memory and search speed scores at 60–64 years and 69 years per 1 *SD* increase in cortical, trabecular and total vBMD and lumbar spine and total hip aBMD at age 60–64 years, adjusted for current height and weight.

	**Search speed**								**Memory**							
	**60–64**				**69**				**60–64**				**69**			
	***N***	**B**	**95% CI**	***p***	***N***	**B**	**95% CI**	***p***	***N***	**B**	**95% CI**	***p***	***N***	**B**	**95% CI**	***p***
**WOMEN**																
**pQCT- 50% radius**																
Cortical vBMD (mg/cm^3^)	684	0.031	−0.042 to 0.104	0.40	601	0.021	−0.060 to.102	0.61	686	−0.030	−0.103 to 0.043	0.41	597	0.011	−0.068 to 0.090	0.78
**pQCT-distal radius**																
Trabecular vBMD (mg/cm^3^)	674	0.063	−0.011 to 0.137	0.09	593	0.029	−0.054 to 0.087	0.48	676	−0.025	−0.100 to 0.049	0.50	589	−0.023	−0.105 to 0.059	0.58
Total Density vBMD (mg/cm^3^)	675	0.076	0.002 to 0.150	0.04	594	0.107	0.025 to 0.189	0.01	677	−0.012	−0.088 to 0.063	0.74	590	−0.058	−0.138 to 0.022	0.15
**DXA aBMD (g/cm**^2^**)**																
Spine L1-L4 aBMD	849	0.021	−0.049 to 0.090	0.56	744	0.0	−0.075 to 0.075	0.99	849	−0.014	−0.083 to 0.056	0.70	740	0.009	−0.066 to 0.084	0.23
Total Hip aBMD	841	0.109	0.033 to 0.185	0.005	737	0.067	−0.016 to 0.149	0.11	841	0.009	−0.068 to 0.086	0.81	733	0.071	−0.011 to 0.152	0.08
**MEN**																
**pQCT- 50% radius**																
Cortical vBMD (mg/cm^3^)	644	0.040	−0.041 to 0.121	0.33	567	−0.404	−0.124 to 0.044	0.34	640	0.051	−0.027 to 0.128	0.20	558	0.083	−0.004 to 0.170	0.06
**pQCT-distal radius**																
Trabecular vBMD (mg/cm^3^)	641	0.065	−0.012 to 0.142	0.09	564	0.018	−0.066 to 0.102	0.67	637	−0.008	−0.082 to 0.067	0.83	555	−0.023	−0.110 to 0.063	0.59
Total Density vBMD (mg/cm^3^)	643	0.028	−0.049 to 0.105	0.47	566	0.070	−0.013 to 0.154	0.09	639	0.058	−0.017 to 0.132	0.13	557	−0.080	−0.166 to 0.007	0.07
**DXA aBMD (g/cm**^2^**)**																
Spine L1-L4 aBMD	777	0.020	−0.054 to 0.095	0.59	687	−0.059	−0.139 to 0.020	0.14	772	−0.009	−0.081 to 0.064	0.81	679	0.035	−0.045 to 0.116	0.38
Total Hip aBMD	767	0.019	−0.060 to 0.098	0.63	679	−0.022	−0.108 to 0.063	0.60	763	0.030	−0.049 to 0.109	0.45	671	−0.003	−0.088 to 0.083	0.94

**Table 5:** Standardized search speed scores at 60–64 years and 69 years per 1 *SD* increase in trabecular and total vBMD and total hip aBMD at age 60–64 years, for women, adjusted for body size, menopause and additionally adjusted for adult lifestyle.

	**Search speed**							
	**60–64**				**69**			
	***N***	**B**	**95% CI**	***p***	***N***	**B**	**95% CI**	***p***
**WOMEN**								
**pQCT-distal radius**								
Total Density vBMD (mg/cm^3^)								
Additionally adjusted for education, occupational class and adult lifestyle	675	0.081	0.007 to 0.154	0.03	588	0.122	0.040 to 0.205	0.004
Additionally adjusted for search speed at 60–64					588	0.079	0.010 to 0.14	0.02
**DXA aBMD (g/cm**^2^**)**								
Total Hip aBMD								
Additionally adjusted for education, occupational class and adult lifestyle	841	0.102	0.026 to 0.178	0.009	730	0.071	−0.012 to 0.155	0.09
Additionally adjusted for search speed at 60-64					730	0.001	−0.068 to 0.070	0.97
**WOMEN SAMPLE WITH AVAILABLE DATA IN MENOPAUSE**								
**DXA aBMD (g/cm**^2^**)**								
Total Hip aBMD	692	0.13	0.046 to 0.217	0.003	608	0.069	−0.024 to 0.162	0.14
Additionally adjusted for menopause	692	0.12	0.035 to 0.209	0.006	608	0.058	−0.037 to 0.153	0.22
Additionally adjusted for education, occupational class and adult lifestyle	692	0.10	0.017 to 0.198	0.02	608	0.057	−0.041 to 0.154	0.25

*In models predicting standardized search speed scores at 69, additionally adjusted for standardized search speed scores at 60–64*.

Hip aBMD was also associated with higher search speed scores at age 60–64 after adjustment for body size (Table 4), and this association was only slightly attenuated after adjustment for educational attainment, smoking, physical activity, and occupational class (Table 5). For women with data on the menopause transition, additional analyses showed that the associations between hip aBMD and search speed at age 60–64 remained after adjustment for type of menopause and timing of period cessation (Table 5).

There were no significant associations between aBMD or vBMD and verbal memory at age 60–64 or age 69 except for a weak positive association between hip aBMD and verbal memory at age 69 (Table 4). This association became stronger after adjustment for the memory score at 60–64 (Table 6).

**Table 6:** Standardized verbal memory scores at 60–64 years and 69 years per 1 *SD* increase in total hip aBMD at age 60–64 years, for women, and trabecular and total vBMD, for men, adjusted for body size and additionally adjusted for education and adult lifestyle.

	**Memory**							
	**60–64**				**69**			
	***N***	**B**	**95% CI**	***p***	***N***	**B**	**95% CI**	***p***
**WOMEN**								
**DXA aBMD (g/cm**^2^**)**								
Total Hip aBMD								
Additionally adjusted for education, occupational class and adult lifestyle	841	−0.014	−0.085 to 0.057	0.69	725	0.062	−0.015 to 0.139	0.11
Additionally adjusted for memory at 60-64					725	0.069	0.005 to 0.132	0.03
**MEN**								
**pQCT-distal radius**								
Trabecular vBMD (mg/cm^3^)								
Additionally adjusted for education, occupational class and adult lifestyle	637	−0.051	−0.120 to 0.018	0.14	548	−0.085	−0.164 to −0.005	0.03
Additionally adjusted for memory at 60-64					548	−0.035	−0.101 to 0.032	0.30
Total Density vBMD (mg/cm^3^)								
Additionally adjusted for education, occupational class and adult lifestyle	639	0.053	−0.015 to 0.122	0.12	550	−0.097	−0.176 to −0.018	0.01
Additionally adjusted for memory at 60–64					550	−0.119	−0.184 to −0.053	<0.001

*In models predicting standardized verbal memory scores at 69, additionally adjusted for standardized verbal memory scores at 60–64*.

In men, there were no associations between BMD and search speed or verbal memory at ages 60–64 and 69 when adjusted for body size only (Table 4). However, associations between greater trabecular and total vBMD with lower verbal memory at age 69 emerged after additionally adjusting for educational attainment, smoking, physical activity, and occupational class (Table 6). The inverse association between total density vBMD and memory at 69 was further strengthened after adjustment for verbal memory scores at ages 60–64 but the association with trabecular vBMD was attenuated (Table 6).

## Discussion

The present study tested associations between cognitive ability in childhood and midlife and bone outcomes at age 60–64; and associations between these bone measures and contemporaneous and subsequent cognitive function.

With regards to childhood cognitive ability and BMD, we found that higher childhood cognitive ability was associated with higher hip aBMD in women, and greater cortical and trabecular vBMD in men. For both men and women, these associations were partly explained by pubertal timing and fully explained after additional adjustment for smoking, leisure time physical activity and occupational class. Further positive associations were found between midlife search speed and trabecular vBMD at age 60–64 in men but not in women. To our knowledge, the present study is the first to investigate associations between prior cognitive ability and bone health later in life, and suggests the possibility of bi-directionality in the association between BMD and cognitive ability; previously reported in studies assuming bone variables as predictors of cognitive ability (Yaffe et al., 1999; Lui et al., 2003). Moreover, these associations were apparently explained by social and behavioral pathways, as Yaffe et al. (1999) suggested. In other words, individuals with greater cognitive ability in their early life are more likely to engage in healthy behaviors (e.g., leisure time physical activity) in their adulthood which are associated with greater BMD later in life (Rianon et al., 2012).

Regarding the associations between BMD and contemporaneous cognitive ability and with cognitive ability 5–9 years later, for women, a positive association was found between greater total vBMD and higher search speed at 60–64 and 69. There was also a positive association between hip aBMD and search speed at 60–64, which was only slightly attenuated by educational attainment, health behaviors and occupational class. A positive association between hip aBMD and verbal memory at age 69 was also found which was strengthened by adjustment for memory score at 60–64, suggesting an association with memory decline over these years. Although not directly comparable due to the different cognitive and bone measures examined, these results are generally consistent with previous cross-sectional research (Zhang et al., 2001; Brownbill and Ilich, 2004; Lee et al., 2012), and are in line with longitudinal studies showing a positive association between BMD and cognitive performance or decline (Yaffe et al., 1999; Lui et al., 2003; Tan et al., 2005; Zhou et al., 2011; Sohrabi et al., 2015). Future studies examining associations between BMD and decline in a wider range of cognitive measures than those in the present study are needed.

We should highlight that the most consistent associations between cognitive ability and bone health in women were found for hip aBMD. BMD and cognitive ability are already known to be independent predictors of a wider range of health outcomes, and their consistent association in our study provides further evidence of their role as indicators of general health. Moreover, these results are relevant from epidemiological and clinical perspectives, as hip aBMD is a well-known predictor of hip fractures, which in turn are associated with higher morbidity and earlier mortality in older adults (Keene et al., 1993; Magaziner et al., 2000).

Previous observations of associations between BMD and cognitive ability have been attributed to the potential role of estrogen, supported by sex differences in this association in older adults (Tan et al., 2005; Sohrabi et al., 2015). Menopausal timing (an indicator of estrogen exposure) did not account for the associations between hip aBMD and search speed. Our findings are consistent with the studies that found similar results whether women taking hormone replacement therapy were included or excluded (Yaffe et al., 1999; Lui et al., 2003). Other possible physiological common cause mechanisms include cortisol and adrenal androgens, which can influence BMD (Hardy and Cooper, 2010) and, in the case of cortisol, impair cognition (Lupien et al., 2007).

In men, there were fewer associations between BMD and cognitive ability at 60–64 and 69, and these only emerged when education, occupational class, smoking and physical activity were included, suggesting that these covariables were masking underlying associations. Lower trabecular and total vBMD were associated with higher verbal memory at age 69, and total vBMD was associated with faster decline in memory from 60–64 to 69 years. Although these results are apparently inconsistent with previous research on men only samples, these are not directly comparable since the present study is the first, to our knowledge, to include volumetric bone measures in this context.

A key strength of this study is the availability of prospective data on cognitive development, which provides the unique opportunity to examine its relationship with subsequent BMD and vice versa. Our findings highlight the need for a life course approach to this topic, to better understand the reciprocal relationships between these factors. As Kuh (2007) highlights, this life course approach which investigates how biological and social factors from early life influence peak levels of function at maturity and their subsequent rate of change, encourages the testing of possible common cause hypotheses at the individual, system or cellular levels, that may eventually translate into frailty late in life (Kuh, 2007; Howlett and Rockwood, 2013).

A further strength is that bone health was assessed at a similar age in all participants, eliminating the need to address age heterogeneity. However, a study limitation is the lack of previous measures of bone health, including during development; thus we cannot be sure when in the life course a bone-cognition association develops, and whether such an association is acting in a specific direction or results from common cause or shared risk factors. Since peak bone mass, a major determinant of bone mass later in life (Bonjour et al., 2009), is reached toward the end of the second and into the third decade, (Berger et al., 2010) future studies should investigate possible associations between BMD during this period and contemporaneous and subsequent cognitive function. Following this, further research on early life determinants of the long-term impact of physical activity for peak bone mass is needed. Our results suggest that individuals with greater cognitive scores in childhood might be more likely to be physically active in their first decades of life, which could contribute to greater peak bone mass at the end of the second and third decade, which in turn would be associated with greater BMD later in life and potentially reduce their rate of bone loss later in life. Moreover, numerous studies have explored the association between higher micronutrient intake (e.g., calcium) and better vitamin D status in childhood and BMD in adulthood (Nieves, 2005; Bonjour et al., 2009). Although the 1946 birth cohort does not have childhood nutrition data or vitamin D status measurements at the micronutrient level at the time cognition was assessed, previous research in this cohort showed that food and nutrient intakes in the 50s were adequate, despite of the relative austerity of post-war supplies, and arguably closer to current recommendations on healthy eating than in the 90s (Prynne et al., 1950). Future research examining the role of micronutrients intake and vitamin D status in childhood and later BMD and cognitive function at the population level, considering potential cohort effects would be of great interest.

In sum, our study suggests that associations between bone health and cognitive ability are best understood within a life course framework. Considering that low BMD is a well-known predictor of fracture, which is a major cause of disability among older adults, the potential role of behavioral factors as smoking and physical activity should be addressed when advising adults at high future risk of osteoporosis and fracture from a public health perspective.

## Author contributions

RB, DK, and MR substantially contributed to the conception and design of the work; analyzing and interpreting the data; and, drafting and critically revising for important intellectual content. DK, MR, JA, and RC substantially contributed to the acquisition of the data. SM, RC, GM, and KW contributed with the interpretation of the data. All the authors critically revised the manuscript for important intellectual content, approved the final version to be published and agreed to be accountable for all aspects of the work in ensuring that questions related to the accuracy or integrity of any part of the work are appropriately investigated and resolved.

### Conflict of interest statement

The authors declare that the research was conducted in the absence of any commercial or financial relationships that could be construed as a potential conflict of interest.

## References

[B1] Berger C. Goltzman D. Langsetmo L. Joseph L. Jackson S. Kreiger N.. (2010). Peak bone mass from longitudinal data: implications for the prevalence, pathophysiology, and diagnosis of osteoporosis. J. Bone Miner. Res. 25, 1948–1957. 10.1002/jbmr.95 20499378 PMC5101070

[B2] Bliuc D. Nguyen N. D. Alarkawi D. Nguyen T. V. Eisman J. A. Center J. R. (2015). Accelerated bone loss and increased post-fracture mortality in elderly women and men. Osteoporos. Int. 26, 1331–1339. 10.1007/s00198-014-3014-9 25600473

[B3] Bonjour J. P. Chevalley T. Ferrari S. Rizzoli R. (2009). The importance and relevance of peak bone mass in the prevalence of osteoporosis. Salud Publica Mex. 51, s5–s17. 10.1590/s0036-36342009000700004 19287894

[B4] Brownbill R. A. Ilich J. Z. (2004). Cognitive function in relation with bone mass and nutrition: cross-sectional association in postmenopausal women. BMC Womens Health 4:2. 10.1186/1472-6874-4-2 15163349 PMC434519

[B5] Cole T. J. (2000). Sympercents: symmetric percentage differences on the 100 loge scale simplify the presentation of log transformed data. Stat. Med. 19, 3109–3125. 10.1002/1097-0258(20001130)19:22<3109::AID-SIM558>3.0.CO;2-F 11113946

[B6] Cole T. J. Kuh D. Johnson W. Ward K. A. Howe L. D. Adams J. E.. (2016). Using Super-Imposition by Translation And Rotation (SITAR) to relate pubertal growth to bone health in later life: the Medical Research Council (MRC) National Survey of Health and Development. Int. J. Epidemiol. 45, 1125–1113. 10.1093/ije/dyw134 27466311 PMC5841778

[B7] Dong Y. Peng C. Y. J. (2013). Principled missing data methods for researchers. Springerplus 2:222. 10.1186/2193-1801-2-222 23853744 PMC3701793

[B8] Egan M. Jaglal S. Byrne K. Wells J. Stolee P. (2007). Factors associated with a second hip fracture: a systematic review. Clin. Rehabil. 22, 272–282. 10.1177/0269215507081573 18057086

[B9] Frost S. A. Nguyen N. D. Center J. R. Eisman J. A. Nguyen T. V. (2013). Excess mortality attributable to hip-fracture: a relative survival analysis. Bone 56, 23–29. 10.1016/j.bone.2013.05.006 23684802

[B10] Galobardes B. Shaw M. Lawlor D. A. Lynch J. W. Davey Smith G. (2006). Indicators of socioeconomic position (part 2). J. Epidemiol. Commun. Health 60, 95–101. 10.1136/jech.2004.028092 16415256 PMC2566160

[B11] Haentjens P. Magaziner J. Colón-Emeric C. S. Vanderschueren D. Milisen K. Velkeniers B.. (2010). Meta-analysis: excess mortality after hip fracture among older women and men. Ann. Intern. Med. 152, 380–390. 10.7326/0003-4819-152-6-201003160-00008 20231569 PMC3010729

[B12] Hardy R. Cooper M. S. (2010). Adrenal gland and bone. Arch. Biochem. Biophys. 503, 137–145. 10.1016/j.abb.2010.06.007 20542010

[B13] Howlett S. E. Rockwood K. (2013). New horizons in frailty: ageing and the deficit-scaling problem. Age Ageing 42, 416–423. 10.1093/ageing/aft059 23739047

[B14] Johnell O. Kanis J. A. Oden A. Sernbo I. Redlund-Johnell I. Petterson C.. (2004). Mortality after osteoporotic fractures. Osteoporos. Int. 15, 38–42. 10.1007/s00198-003-1490-4 14593451

[B15] Keene G. S. Parker M. J. Pryor G. A. (1993). Mortality and morbidity after hip fractures. BMJ 307, 1248–1250. 10.1136/bmj.307.6914.1248 8166806 PMC1679389

[B16] Kuh D. (2007). A life course approach to healthy aging, frailty, and capability. J. Gerontol. A Biol. Sci. Med. Sci. 62, 717–721. 10.1093/gerona/62.7.717 17634317

[B17] Kuh D. Muthuri S. Cooper R. Moore A. MacKinnon K. Cooper C.. (2016b). Menopause, reproductive life, hormone replacement therapy and bone phenotype at age 60–64: a British birth cohort. J. Clin. Endocrinol. Metab. 101, 3827–3837. 10.1210/jc.2016-1828 27472291 PMC5052353

[B18] Kuh D. Muthuri S. G. Moore A. Cole T. J. Adams J. E. Cooper C.. (2016a). Pubertal timing and bone phenotype in early old age: findings from a British birth cohort study. Int. J. Epidemiol. 45, 1–20. 10.1093/ije/dyw131 27401728 PMC5075580

[B19] Kuh D. Pierce M. Adams J. Deanfield J. Ekelund U. Friberg P.. (2011). Cohort profile: updating the cohort profile for the MRC National Survey of Health and Development: a new clinic-based data collection for ageing research. Int. J. Epidemiol. 40, e1–e9. 10.1093/ije/dyq231 21345808 PMC3043283

[B20] Kuh D. Wills A. K. Shah I. Prentice A. Hardy R. Adams J. E.. (2014). Growth from birth to adulthood and bone phenotype in early old age: a British birth cohort study. J. Bone Miner. Res. 29, 123–133. 10.1002/jbmr.2008 23761289 PMC4292430

[B21] Lee D. Y. Na D. L. Seo S. W. Chin J. Lim S. Choi D.. (2012). Association between cognitive impairment and bone mineral density in postmenopausal women. Menopause 19, 636–641. 10.1097/gme.0b013e31823dbec7 22334060

[B22] Little R. J. A. Rubin D. B. (2002). Statistical Analysis with Missing Data, 2nd Edn. New York, NY: Wiley.

[B23] Lui L. Y. Stone K. Cauley J. A. Hillier T. Yaffe K. (2003). Bone loss predicts subsequent cognitive decline in older women: the study of osteoporotic fractures. J. Am. Geriatr. Soc. 51, 38–43. 10.1034/j.1601-5215.2002.51007.x 12534843

[B24] Lupien S. J. Maheu F. Tu M. Fiocco A. Schramek T. E. (2007). The effects of stress and stress hormones on human cognition: implications for the field of brain and cognition. Brain Cogn. 65, 209–237. 10.1016/j.bandc.2007.02.007 17466428

[B25] Magaziner J. Hawkes W. Hebel J. R. Zimmerman S. I. Fox K. M. Dolan M.. (2000). Recovery from hip fracture in eight areas of function. J. Gerontol. A Biol. Sci. Med. Sci. 55, M498–M507. 10.1093/gerona/55.9.m498 10995047

[B26] Marshall D. Johnell O. Wedel H. (1996). Meta-analysis of how well measures of bone mineral density predict occurrence of osteoporotic fractures. BMJ 312, 1254–1259. 10.1136/bmj.312.7041.1254 8634613 PMC2351094

[B27] Melton L. J. Atkinson E. J. O'Connor M. K. O'Fallon W. M. Riggs B. L. (1998). Bone density and fracture risk in men. J. Bone Miner. Res. 13, 1915–1923. 10.1359/jbmr.1998.13.12.1915 9844110

[B28] Nieves J. W. (2005). Osteoporosis: the role of micronutrients. Am. J. Clin. Nutr. 81, 1232–1239. 15883457 10.1093/ajcn/81.5.1232

[B29] Pigeon D. A. (1968). Detail of the fifteen years tests, in All Our Future, eds Douglas J. W. B. Ross J. M. Simpson H. R. (London: Davies), 1.

[B30] Prynne C. J. Paul A. A. Price G. M. Day K. C. Hilder W. S. Wadsworth M. E. J. (1950). Food and nutrient intake of a national sample of 4-year-old children in: comparison with the 1990s. Public Health Nutr. 2, 537–547. 10656473 10.1017/s1368980099000725

[B31] Rianon N. J. Lang T. F. Sigurdsson G. Eiriksdottir G. Sigurdsson S. Garcia M.. (2012). Lifelong physical activity in maintaining bone strength in older men and women of the Age, Gene/environment susceptibility-Reykjavik study. Osteoporos. Int. 23, 2303–2312. 10.1007/s00198-011-1874-9 22234811 PMC4940059

[B32] Richards M. Sacker A. (2003). Lifetime antecedents of cognitive reserve. J. Clin. Exp. Neuropsychol. 25, 614–624. 10.1076/jcen.25.5.614.14581 12815499

[B33] Sohrabi H. R. Bates K. A. Weinborn M. Bucks R. S. Rainey-Smith S. R. Rodrigues M. A.. (2015). Bone mineral density, adiposity, and cognitive functions. Front. Aging Neurosci. 7:16. 10.3389/fnagi.2015.00016 25741279 PMC4332358

[B34] Stafford M. Black S. Shah I. Hardy R. Pierce M. Richards M.. (2013). Using a birth cohort to study ageing: representativeness and response rates in the National Survey of Health and Development. Eur. J. Ageing 10, 145–157. 10.1007/s10433-013-0258-8 23637643 PMC3637651

[B35] Tan Z. S. Seshadri S. Beiser A. Zhang Y. Felson D. Hannanm M. T.. (2005). Bone mineral density and the risk of Alzheimer disease. Arch. Neurol. 62, 107–111. 10.1001/archneur.62.1.107 15642856

[B36] Yaffe K. Browner W. Cauley J. Launer L. Harris T. (1999). Association between bone mineral density and cognitive decline in older women. J. Am. Geriatr. Soc. 47, 1176–1182. 10.1111/j.1532-5415.1999.tb05196.x 10522949

[B37] Zhang Y. Seshadri S. Ellison R. C. Heeren T. Felson D. T. (2001). Bone mineral density and verbal memory impairment: third National Health and Nutrition Examination Survey. Am. J. Epidemiol. 154, 795–802. 10.1093/aje/154.9.795 11682361

[B38] Zhou R. Deng J. Zhang M. Zhou H. D. Wang Y. J. (2011). Association between bone mineral density and the risk of Alzheimer's disease. J. Alzheimers. Dis. 24, 101–108. 10.3233/JAD-2010-101467 21187587

